# Procedure to Estimate Added and Free Sugars in Food Items from the Swedish Food Composition Database Used in the National Dietary Survey Riksmaten Adolescents 2016–17

**DOI:** 10.3390/nu11061342

**Published:** 2019-06-14

**Authors:** Julia Wanselius, Cecilia Axelsson, Lotta Moraeus, Christina Berg, Irene Mattisson, Christel Larsson

**Affiliations:** 1 Department of Food and Nutrition, and Sport Science, University of Gothenburg, SE-405 30 Gothenburg, Sweden; christina.berg@ped.gu.se (C.B.); christel.larsson@gu.se (C.L.); 2 The National Food Agency, Sweden, Risk Benefit Assessment department, SE-751 26 Uppsala, Sweden; cecilia.axelsson@slv.se (C.A.); lotta.moraeus@slv.se (L.M.); evairene@live.se (I.M.)

**Keywords:** added sugars, free sugars, sugars, children, adolescents, dietary assessment, nutrition recommendations, dietary guidelines, food composition data, national dietary survey

## Abstract

A high intake of added and free sugars is associated with poor diet quality, caries, and potentially has a role in non-communicable diseases. As a result, dietary guidelines advice limitation. However, there is no standardized method for estimation of added and free sugars in food items and consequently intake is difficult to measure. This study aimed to refine a procedure for sugars estimation and apply it to a Swedish dietary survey on adolescents (Riksmaten Adolescents 2016–17). A national sample of 3099 adolescents in school year 5, 8 and 11 participated (55% girls). Individual dietary intake data from two non-consecutive days was collected retrospectively and used for analysis. A ten-step systematic procedure for estimation of sugars in a Swedish context has been developed by combining two earlier methods, one for estimation of added sugars and one for free sugars. Sugars estimates were made for all food items comprising the survey database. Mainly objective decisions were necessary to make the estimates (92% and 93% for the sugars respectively); meaning that the procedure was largely transparent. In relation to Nordic Nutrition Recommendations, 45% of the participants had an intake that adhered to the guidelines. However, the majority of intakes was close to the recommendation. Further research on how specific food sources contribute to added and free sugars is necessary to facilitate further guidance on sugars and how to reach recommended target levels in Sweden.

## 1. Introduction

Inverse associations between high intake of added and free sugars and micronutrient intake have been observed, and diets rich in sugars have shown to have a lower diet quality [1,2]. High intake of added and free sugars are also related to an increased risk of dental caries [3], and obesity [4], and may potentially be related to diseases such as cardiovascular diseases and type 2 diabetes [5,6]. As a result, dietary guidelines around the world advice a limitation of sugars in the diet. Today, recommendations in Sweden are a maximum intake of 10% of total energy intake from added sugars [7], whereas the World Health Organization (WHO) recommend a maximum of 10% of total energy intake from free sugars, with conditional recommendations to aim for an energy intake from free sugars below 5% [8]. However, added and free sugars content in food items has rarely been published, especially in Europe, limiting the possibility to evaluate sugars consumption in regards to sugars recommendations. There is no existing standardized method for estimation of the content of added and free sugars in food items, consequently comparisons between different studies is difficult and studies may yield diverse results. 

“Sugars” conventionally covers all mono- and disaccharides [7]. Several subdivisions of sugars exist with varying definitions, most frequently known as “added sugars” and “free sugars”. In Europe, added sugars are commonly equated with refined sugars or isolated sugar preparations that are added during cooking or manufacturing [7,9]. Free sugars are all added sugars as well as sugars in honey, syrup, fruit juice and fruit juice concentrate [8]. However, definitions of added and free sugars vary between different guidelines, creating different categorical definitions. 

Sugars added to foods and sugars naturally occurring in foods cannot be differentiated by their molecular structure as they do not differ. Consequently, there are no analytical laboratory methods to determine if sugars are added or not. On nutrition declarations of food items, the declared information is commonly based on available chemical measurements for sugars; i.e., total sugars. Total sugars, as implied by its name, is the total sugars content in foods; added as well as naturally occurring. Thereby the consumers are not informed of how much of the sugars in a food item are added or free. 

The development of reliable and transparent sugars estimation methods is necessary to evaluate, monitor and compare sugars consumption between populations. Louie et al. modelled a starting point for standardized sugars estimations in 2014 when they proposed a systematic procedure for estimating added sugars in Australian food items [10]. The method provided good repeatability for added sugars estimations. Correspondingly, in 2017, Kibblewhite, Nettleton et al., applied the procedure described by Louie et al. with modifications to provide free sugars estimates in New Zealand food items and population [11].

The overall objective of this study was to refine a procedure for estimating the content of added and free sugars to be used in the Swedish food composition database and to be implemented on a national Swedish dietary survey on adolescents’ food intake (Riksmaten Adolescents 2016–17). The sugars content estimation procedure is based on the methods previously described by Louie et al. [10], and Kibblewhite, Nettleton et al [11]. The present study aimed to refine the estimation procedure to include more food items than previous procedures, to broaden the applicability on different dietary patterns. Furthermore, the aim was to provide estimates of the amount of added and free sugars consumed by Swedish adolescents.

## 2. Materials and Methods 

Added and free sugars content was estimated for food items from the Swedish food composition database, administered by the National Food Agency, Sweden (NFA), used in the Riksmaten Adolescents 2016–17 survey. The sugars content estimation procedure was developed by combining and refining procedures for estimation of added sugars described by Louie et al. [10], and free sugars described by Kibblewhite, Nettleton et al. [11]. The primarily principle in this procedure is to base the estimations on information of total and naturally occurring sugars in individual food items. Estimations of added and free sugars were inferred from total sugars, which were calculated as the sum of total mono- and disaccharides in all food items. The new database values for added and free sugars were then used together with food intake data to calculate intake in Swedish adolescents. 

Added sugars were defined in accordance to the Nordic Nutrition Recommendations 2012 (NNR 2012) and European Food Safety Authority (EFSA) [7,9] as being sugars from all food items where refined sugars is added during cooking or manufacturing, not including honey or unsweetened fruit juices. Free sugars were defined according to WHO’s definition [8]; sugars from all food items containing added sugars, as well as sugars naturally present in honey, syrups, fruit juice and fruit juice concentrate. Sugars from vegetable juices were not included. 

### 2.1. Participants

In the fall of 2016 and spring of 2017, the NFA carried out the dietary survey Riksmaten Adolescents 2016–17 on a national representative sample of adolescents in school year 5, 8 and 11 (mean ages 12, 15 and 18 years old respectively) [12]. The study is described in detail by Moraeus et al. [13]. Briefly, the study was conducted class-wise in schools randomly selected from the Swedish school register administrated by Statistics Sweden. Sampling was based on school size, geographic area, and municipality characteristics (such as population and commuting patterns). The participating schools covered geographical areas across the country, with participants representative to the Swedish population in regard to socioeconomic background and school organization. Out of 5145 adolescents invited, 3099 participated with full dietary information. The participant distribution was 34% year-5 students (53% girls), 34% year-8 students (55% girls), and 32% year-11 students (58% girls)

All participants and their legal guardian received information about the study one month prior the start, and could withdraw from the study at any time without giving any reason. The participants gave their informed consent for inclusion when they participated in the study. The study was conducted in accordance with the Regional Ethical Review Board in Uppsala (No. 2015/190).

### 2.2. Dietary Assessment

Individual dietary intake data from two non-consecutive days, as recommended by EFSA for estimation of habitual population intake distribution [14], were used. The first day of retrospective registration was always scheduled to be the day prior to the initial school visit from the NFA, when the method was presented and the survey described. The second day of retrospective registration was randomly assigned each participant, aiming for a representative distribution of weekdays (Monday through Thursday) and weekends (Friday through Sunday). Dietary intake was assessed both in spring and in autumn to capture seasonal variations.

Information on dietary intake was collected through a newly developed web-based food assessment method, “RiksmatenFlexDiet”. RiksmatenFlexDiet is a digital 24-h recall, where the participants registered their intake retrospectively by selecting the food items they had consumed. In a first step, the participants selected what food items they had eaten from a food list containing 778 items. In a second step they could specify the food—i.e., if they had eaten fish soup, they specified type of fish and soup base. They also specified their intake amount in either standard portions sizes, pieces, household measurements or through portion pictures, which resulted in gram values for each selected food item. 

### 2.3. Food Items Used in the Riksmaten Adolescents 2016–17 Survey

All food items used in the Riksmaten Adolescents 2016–17 survey were retrieved from the Swedish food composition database. Food items in the database are either single food items or composite food items. Nutrient values in single food items are mainly determined through laboratory analysis, but they can also be estimated from similar food items, product information, or from the non-occurrence of naturally present nutrients (logical zero). Composite food items are composed of two or more food items, single and/or composite. For example, remoulade is a composite food item constructed in two stages:

(1) Remoulade contains the single food items pickles, parsley and chives; and it contains the composite food item mayonnaise.
(2) Mayonnaise only contains single food items (oils, vinegar, egg yolk, lemon juice, table salt, French mustard).

Hence, all food items originate from single food items, and both single and composite items can appear in several composite food items.

In the Riksmaten Adolescents 2016–17 survey, the participants could search from the food list of 778 food items, selected from the Swedish food composition database. The food list was based on statistics on food intake in the age groups as well as target group interviews. In order to facilitate the participants in finding the correct individual food item, it was decided that mostly generic food items should be included. As several food items were included in the composite items, the 778 items selected originated from 1483 items (844 single and 639 composite) from the Swedish food composition database. By estimating the sugars content in the 844 single food items included, sugars content in the composite items could automatically be calculated according a standardized recipe calculation method [15]. 

### 2.4. Procedure to Estimate Added and Free Sugars Content in Food Items

**Figure 1:**
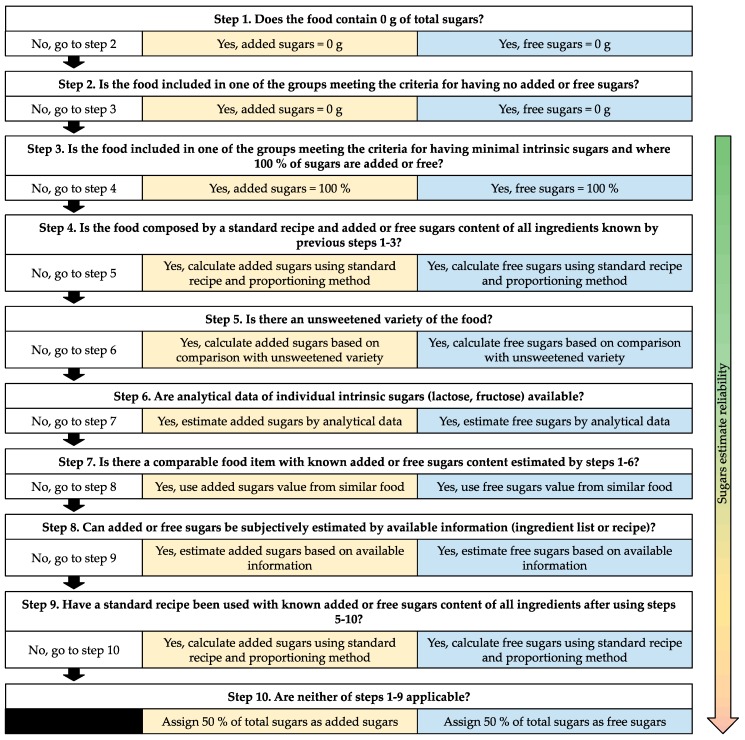
Ten-step decision-making procedure for estimating added and free sugars content in food items. Yellow boxes (middle column) indicate stepwise decision for added sugars, blue boxes (right column) for free sugars. The refined procedure is based on two previously described procedures by Louie et al. [10] and Kibblewhite, Nettleton et al. [11].

The sugars estimation procedure works in a stepwise manner from 1 to 10, estimating each individual food item’s added and free sugars content based on the item’s available sugars content data, categorizing them in food groups within the different steps. The ten-step procedure consists of six steps of objective decisions (1–6), and four steps of subjective decisions (7–10). The highest reliability of sugars estimation accuracy is achieved in the lowest step numbers (where given less room for subjective decision). The two procedures described by Louie et al. [10] and Kibblewhite, Nettleton et al. [11], were not identical in their structure of step allocation. Agreement between the steps were made in order to let the same food item fall into the same step when estimating either added or free sugars (where possible), i.e., consistent steps were created within the new model (results presented in Figure 1, with detailed information in Appendix A). In contrast to the previous publications, honey was excluded from the definition of added sugars in compliance with the Nordic Nutrition Recommendations (NNR 2012) [7], and fruit purées excluded from the free sugars definition according to recommendations by the WHO [8], which led to differences in food categorization. 

Before added and free sugars estimates were made, food items common in a Swedish setting were found not be included within any of the steps. Sweetened, cured and pickled products, which are traditional in the Swedish cuisine (e.g., gravlax, pickled herring and pickled vegetables), were added as a separate category. In addition, since breads in Sweden often are sweetened with sugars, rather than only using sugars to activate yeast for fermentation, plain breads were described and approached differently. Instead of categorizing all plain breads (except gluten free) as non-sugars containing, as done in the two other described procedures [10,11], all breads were checked for sugars content. Minimal amounts of sugars in bread were discounted (<9 g/>1000 g pre-baking weight). Further modifications included alterations in food categories and additions of food items within the procedure steps during the course of work when food items appeared not to belong to any of the groups within the steps, to broaden the applicability of the procedure. This was mostly due to traditional national recipes and variation in composition of dishes. Additions in food categories included a new category for pseudocereals (i.e., non-grass plants with starchy seeds commonly used in the same way as cereals, e.g., buckwheat, quinoa and amaranth). Additions in food items included non-dairy milk alternatives other than soy products (e.g., oat and rice products), blood products, and mushrooms. A full list of dissimilarities between the refined procedure and the two other procedures are found in Appendix B.

Changes were made in the procedure for calculations when food items contained fructose (step 6), and for calculations of the proportional amount of sugars (step 8). In step 6, available analytical data on fructose was used (see steps 6 b and c in Appendix A). Fructose was subtracted from total sugars in food items containing fruits and/or vegetables. Steps 6 b and c was applied on 10 items covering baked goods with fruits, breakfast cereals and Swedish hash. As only fructose was subtracted from total sugars, other intrinsic sugars were not accounted for.

### 2.5. Estimating Added and Free Sugars Content in Food Items in the Swedish Food Composition Database

Added and free sugars content estimates were systematically assigned all 1483 food items forming the food list following the refined 10-step procedure. During a period of two weeks, one of the authors (J.W.) made added and free sugars content estimates. Estimations were based on information available on Swedish food products. If the exact type of product was not specified within the food composition database, estimations were based on an average of three or more popular food items from popular supermarkets [16]. The information was collected from declared values presented on the label of the food items concerned. This was collected online, either from supermarkets’ or particular brands’ official websites. When estimating sugars in ready-made dishes where additional information was needed on recipe proportions, cookbook recipes from Sweden’s highest selling basic cookbook were used [17,18]. Steps 1–3, 5–8, and 10 were used to estimate sugars content in single food items and the remaining two steps were used for composite food items. If composite food items were only comprised of food items assigned steps 1–4, they were assigned step 4. If composite food items contained at least one food item assigned step 5–10, the composite food item was assigned step 9. The estimates were proposed to food composition data experts (C.A. and I.M. at the NFA) during meetings held twice daily during a period of ten days in order to discuss any issues that arose. When sugars content estimates had been assessed for all food items, the estimates were inspected by two additional researchers and later entered into the Swedish food composition database. As a final step, the sugars estimate values added into the database were double-checked by the author who proposed the values. 

### 2.6. Statistical Methods

As the dietary intakes were assessed over two days per participant, the data is insufficient to account for day-to-day variation on an individual level. To convert the short-term intake data to habitual (long-term average) intake, the multiple source method (MSM) was used. The MSM is a web-based application for estimation of usual daily dietary intakes based on short term measurements [19], (http://nugo.dife.de/msm). Sugars and energy intake values from the two reported intake days were adjusted to calculate individual habitual daily intakes using the MSM. All participants in this study were assumed to be daily consumers of both added and free sugars.

To obtain percent of energy for added and free sugars, the individual habitual sugars and energy values from the MSM estimations were used. Median percent of energy from added respective free sugars was calculated stratified on sex and school year, Mann–Whitney U-test were used to test for differences between sexes within the same strata.

Statistical analyses were performed using IBM SPSS Statistics version 25.0 (IBM Corp., Armonk, NY, USA).

## 3. Results

### 3.1. Estimates of Added and Free Sugars Content in the Swedish Food Composition Database

For added sugars, 92% of the food items were assigned an objective step number (steps 1–6), and 8% a subjective (steps 7–10). For free sugars, 93% were assigned an objective step number, and 7% a subjective. This means that the procedure was largely transparent. Complete spreading of step number assignments can be viewed in Table 1. Of the food items, 521 (35%) contained added sugars, and 585 (39%) contained free sugars.

### 3.2. Estimates of Intakes of Added and Free Sugars in Swedish Adolescents

In total, 45% of the participants had intakes of added sugars below 10% of total energy intake, complying with the current Nordic Nutrition Recommendations [7]. For free sugars, 30% had intakes below 10% of total energy intake, and 3% had intakes of free sugars below 5% (intakes of sugars presented in Table 2). Girls in all school years had higher intakes of sugars as a percentage of total energy intake of all types of sugars compared to boys in the same school year (*p* < 0.05, Mann–Whitney U test).

## 4. Discussion

### 4.1. Estimation of Sugars Content and Intake

Systematic estimates were created for all food items included in and constructing the food items used in Riksmaten Adolescents 2016–17 survey. This was done in accordance with the refined 10-step sugars estimation procedure, based on two previous procedures by Louie et al. [10] and Kibblewhite, Nettleton et al. [11]. The previous procedures were convenient, but adjustments were needed to extend the applicability to various food cultures and for inclusion of different aspects related to Swedish cuisine. Another substantial difference from the two previous procedures is the definition of added sugars only including refined sugars. Merely a limited amount of subjective evaluation of food items was required in the refined procedure, 8% for added sugars and 7% for free sugars. This makes the procedure a straightforward method to estimate objective sugars content without requiring an in-depth knowledge of the composition of individual food items (for most food items), as concluded by Louie et al. [10]. Since there are no analytical methods for differentiating if sugars are added, free, or from other sugars sources, reliable sugars estimation methods with little room for subjectivity are essential. 

Restrictions in sugars consumption is a target for worldwide public health recommendations. However, these recommendations are difficult for consumers to follow due to lacking information in food labelling, with no differentiation between total, added or free sugars. Another problematic aspect is that sugars are being defined differently. In Europe, Nordic countries included, added sugars are commonly equated with refined sugars [7,9]. Even within the Nordic countries, however, it is possible to identify differences in definitions of added sugars. For example, the Keyhole label for guidance on healthier foods administered by the NFA include the following in its definition of added sugars; sugars added to foods from honey, fruit drinks, fruit juices and fruit concentrate [20]. This definition is similar to the WHO’s definition of free sugars. The question in definitions of added sugars is whether added sugars only includes refined sugars or if sugars added during cooking or manufacturing from honey, fruit juices and dried fruits are included, as well. The definition of free sugars introduced by the WHO is clearer. The WHO defines free sugars as all mono- and disaccharides added to foods by the manufacturer along with sugars naturally present in honey, syrups, fruit juices and fruit juice concentrates [8]. The consistent distinction between added and free sugars is that free sugars always includes all sugars from fruit juices and fruit juice concentrates, not only added ones. In this study, a strict definition of added sugars has been used for estimation of sugars content in foods. Other definitions would have classified certain free sugars as added sugars. Inconsistent sugars definitions, and accordingly recommendations, together with unstandardized nutrient databases hinder useful generalisation in research examining health effects related to intake of specific types of sugars, and will yield discrepancies in associations to health outcomes.

Dietary data was collected with a new web-based method inspired by traditional 24-h recalls, where the participants retrospectively recalled dietary intake from the day prior to registration. The method facilitated retrospective registration of actual intake over two days, was relatively non-burdensome for the participants and collected data on dietary habits of a large group of adolescents. Limitations of the method are its reliance on each individual’s memory and ability to visualize portion size, which risks participants not reporting actual dietary intake due to lacking memory or to ease registration [21]. To aid registration, portion pictures complemented most food items and probing questions of food items easily forgotten were set during and at end of registrations. Another limitation could be that even though food items within the survey were carefully chosen to include a span of food items with several variations in portioning, options were limited. Sugars consumption may therefore be underreported. Further, analyzed fructose data in food items containing vegetables and/or fruits were included in one of the steps (step 6) for objective estimation of sugars content. This enabled additional food items with sugars values estimated by an objective step number, using precise measurements on sugars content. Although, since fructose was the only intrinsic sugar type to be subtracted from total sugars, other intrinsic sugars were not accounted for. Here, ten food items mainly containing small amounts of intrinsic sugars fell into the step, giving the items reliable estimates, although estimates can be slightly overestimated.

One of the two registration days was randomly assigned to the participants, aiming for a representative distribution between weekdays (Monday through Thursday) and weekends (Friday through Sunday). This was due to an assumption that food items with high levels of added and free sugars are more frequently consumed during the weekends. The distribution of days reported were 56% on weekdays (Monday through Thursday), and 44% on weekends (Friday through Sunday), hence almost identical to how the seven days in a week are distributed. The distribution of days within the school year and sex strata was comparable. The application of the MSM enabled adjustment of the dietary data from the two reported days to habitual intake. This was beneficial as the number of participants with excessive or low intakes may have been overrepresented otherwise, due to the tails of the distribution being too wide. The limited amount of days reported per participant would not affect the raw data average but could have a considerable effect on habitual intake. An important part of the MSM is to estimate the proportion of habitual consumers of nutrients, wherein this study all participants were assumed to be habitual consumers of both added and free sugars. Only two of the 3099 participants reported no intake of added or free sugars on any of the two days reported, before application of the MSM. This means that the assumption that all participants are habitual sugars consumers holds, although it may be wise to apply some caution with this method when considering nutrients less frequently consumed. 

### 4.2. The Majority Consumed More than Recommended

The representative sample of Swedish adolescents in school years 5, 8 and 11 in this study has provided new insights about sugars consumption of young people. The current NNR 2012 guidelines for sugars consumptions recommend limiting added sugars intake and to have added sugars intake not exceed 10% of total energy intake [7]. Median intakes in this study were 10% of total energy intake for added sugars with the 75th percentile not exceeding 13%. Although only 45% of the population in this study reached the recommended goal of added sugars intake, the majority of the population reported intakes of added sugars close to the recommended. Thus, reaching recommended added sugars intake levels should be possible for Swedish adolescents. Concerning free sugars, 30% of the participants met the WHO guidelines of maximum 10% of free sugars in total energy intake, with only 3% meeting the conditional recommendation of a maximum of 5%. Having this population reach the recommended intake of free sugars as 10% of total energy intake seems possible, as well, since three quarters of the population reported intakes of free sugars at maximum 15% of total energy. However, it should be noted that the intakes probably are underestimated due to selection and social desirability bias, as in other dietary surveys.

Large population based dietary surveys in Sweden are rare, with no previous dietary studies in representative Swedish adolescent samples, thus comparison to similar Swedish studies are inaccessible. However, in earlier Riksmaten population surveys on children (data collected 2003) [22] and adults (data collected 2010–2011) [23], there are approximations of added sugars intake in children (*n* = 2495) of four years of age, in school year 2 and in school year 5; and in adults (*n* = 1791) aged 18–80 years old. In children, average added sugars intake were approximated to be 13–15% of total energy intake, with no differences in added sugars consumption observed between sexes [22]. In adults, average added sugars intake were approximated to be 9.6% of total energy intake, with women reporting less added sugars than men [23]. Although, intake where higher in the lowest age span (18–30 years old) with median intakes approximated to 11% of total energy intake, where women reported highest intake of added sugars of 12% of total energy intake [23]. Another Swedish cross-sectional health survey explored consumption of food items high in sugars among adolescents aged 15–16 years old in a south western part of Sweden in 2008 (*n* = 8308) [24]. This study revealed that boys had higher intakes of food items rich in sugars, and in intake of sucrose as percent of total energy (added sugars consumption was not presented). In the European multicenter cohort study ‘Identification and prevention of dietary and lifestyle induced health effects in children and infants’ (IDEFICS), Swedish children aged 2–9 years old participated. From dietary data collected 2007–2008 (*n* = 8308, of which 14% Swedish participants), free sugars intake was assessed [25]. On average, children had an intake of added sugars of 18% of total energy intake, where no differences were observed between sexes [25]. In our study, girls had higher intakes of sugars than boys, in terms of percent of total energy intake, which contradicts most previous findings. In addition, intakes of sugars are low compared to previous findings, both for added and free sugars. This may be due to varying methods and accuracy in sugars content estimations or different definitions used, because of different populations studied, or may be because a decrease in consumption of sugars rich foods during the last years.

### 4.3. Continous Updates Necessary 

Recommendations on limiting sugars intake aim to minimize health risks associated with sugars, but discrepancies in definitions and subsequent recommendations may yield discrepancies in regards to health outcomes. Even though the molecules within sugars are the same, there are important differences in relation to health outcomes between different food sources. Food items with high levels of free sugars, in contrast to food items with high levels of added or total sugars, can be more clearly related to health risks in regard to caries, energy intake, weight gain and type 2 diabetes [26]. As there is no scientific evidence for a differentiation between sugars in processed fruits or vegetables, processed vegetable sugars should be included in the free sugars definition [27], in contrast to the WHO definition of free sugars that only include sugars from processed fruits. The ten-step systematic procedure used in this study will continuously be used at the NFA to estimate sugars contents in the Swedish food composition database, and to facilitate monitoring and examination of how the Swedish population adheres to sugars recommendations. Sugars from vegetable juices will from now on be included to the free sugars definition at the NFA. Further, the NFA will pay attention to food product development and use of ingredients for future adjustment of the sugars estimate procedure to extend accuracy. This is now easily achievable because of the detailed systematic procedure adapted.

This study did not draw any conclusions concerning how specific food sources contribute to added and free sugars intake among adolescents. Further analysis of which food items contribute to added and free sugars in this population is necessary, since more in-depth knowledge is required to facilitate guidance on consumption of sugars in order to achieve the population’s target levels for sugars.

## 5. Conclusions

The refined ten-step sugars estimation procedure presented in this study facilitated reliable and largely objective estimates of added and free sugars content in food items. The procedure is straightforward, and as mainly objective estimates were necessary, it can easily be administered without requiring an in-depth knowledge of food composition. Most Swedish adolescents are consuming too much sugars in comparison to current guidelines on added and free sugars. However, it is important to note that median intakes are not far from target levels. Policies need to address the high consumption of sugars in order to reduce intakes and to create reasonable health promotive actions with appropriate guidance. A deeper knowledge about which food sources contribute most to high levels of added and free sugars is necessary to facilitate guidance on sugars, and furthermore to achieve target levels within the population. A future deeper knowledge of the intake of added and free sugars in various Swedish populations can be obtained by using the refined procedure of the present study.

## Figures and Tables

**Table 1:** Step distribution for estimates of added and free sugars in all food items used in the Riksmaten Adolescents 2016–17 survey.

Step Number	Number of Categorised Food Items (%)	
	Added Sugars	Free Sugars
1	194 (13.1)	194 (13.1)
2	381 (25.7)	356 (24.0)
3	146 (9.8)	177 (11.9)
4	565 (38.1)	569 (38.4)
5	20 (1.3)	20 (1.3)
6	58 (3.9)	58 (3.9)
7	2 (0.1)	2 (0.1)
8	34 (2.3)	28 (1.9)
9	74 (5.0)	70 (4.7)
10	9 (0.6)	9 (0.6)
Total number of foods	1483 (100)	1483 (100)

**Table 2:** Added and free sugars median (p25; p75) intakes ^1^ in Swedish adolescents by school year and sex.

School Year ^2^	Sex (*n*)	Added Sugars, g/day	Free Sugars, g/day	Total Sugars, g/day	Added Sugars, E% ^3^	Free Sugars, E% ^3^	Total Sugars, E% ^3^
5	All (1049)	46 (34; 59)	55 (42; 68)	95 (75; 114)	10 (8; 12)	12 (9; 14)	20 (17; 23)
	Girl (559)	46 (34; 57)	55 (43; 68)	94 (75;112)	10 (8; 12)	13 (10; 15)	21 (18; 23)
	Boy (490)	46 (34; 61)	54 (40; 68)	96 (75;116)	10 (7; 12)	11 (8; 14)	20 (16; 23)
8	All (1050)	53 (43; 67)	62 (47; 79)	106 (84; 129)	11 (9; 13)	12 (10; 15)	21 (18; 23)
	Girl (574)	50 (42; 58)	58 (46; 68)	96 (78;113)	11 (9; 12)	12 (10; 15)	21 (18; 23)
	Boy (476)	63 (45; 81)	71 (51; 94)	120 (97;149)	10 (8; 13)	12 (9; 15)	20 (17; 23)
11	All (1000)	54 (38; 72)	61 (44; 81)	102 (80; 127)	10 (8; 13)	12 (9; 15)	19 (16; 22)
	Girl (577)	52 (38; 67)	59 (44; 75)	96 (75;118)	11 (9; 13)	12 (10; 15)	20 (17; 23)
	Boy (423)	59 (39; 80)	67 (45; 92)	112 (87;141)	9 (7; 12)	11 (8; 14)	18 (15; 21)
	All (3099)	51 (38; 65)	59 (44; 75)	100 (79; 124)	10 (8; 13)	12 (9; 15)	20 (17; 23)

^1^ Calculated to estimate habitual dietary intake using the Multiple Source Method (MSM); ^2^ Mean age school year 5, 8, and 11: 12, 15, and 18 years old; ^3^ percent of total energy intake.

## Appendix A

The ten-step decision-making procedure for estimation of added and free sugars is modified from methods described by Louie et al. [10] and Kibblewhite, Nettleton et al. [11]. Food item categorizing differences between added and free sugars are marked in bold.

**Table A1:** Detailed ten-step decision-making procedure for estimating added and free sugars content.

	Added Sugars		Free Sugars
Step 1	Food items containing 0 g of total sugars are assigned 0 g of added sugars	Step 1	Food items containing 0 g of total sugars are assigned 0 g of free sugars
Step 2	Food items in the food groups stated below are unprocessed or minimal processed with no added sugars, and are assigned 0 g of added sugars:	Step 2	Food items in the food groups stated below are unprocessed or minimal processed with no free sugars, and are assigned 0 g of free sugars:
	(a) spices and herbs; **honey** (b) fats and oils (c) plain cereal grains, pseudocereals (e.g., buckwheat, quinoa, amaranth), flour, pasta, rice, and plain cereal products; unsweetened potato chips (d) plain breads with minimal amounts of added sugars only used for activation of yeast in fermentation (<9 g/>1000 g pre-baking weight) (e) plain pastries (e.g., puff pastry, pastry dough) without fillings, and without dried fruits, nuts or chocolate (f) eggs and egg products (excluding egg-based desserts) (g) fresh, frozen, or cooked fruits, berries, vegetables (including salads without dressing) and root vegetables; unsweetened dried fruits (h) fruit and vegetables canned in 100% **fruit juice** or vegetable juice, or in artificially sweetened liquid (i) unsweetened nuts, seeds, coconut and coconut products (j) fresh meat, fresh fish, fresh seafood, tofu, and unsweetened legumes and mushrooms; mixed meat dishes without added sugars (k) coffee, tea and alcoholic beverages unsweetened or artificially sweetened (l) unsweetened milk and dairy products, and non-dairy milk substitutes (e.g., oat and soy drinks and yoghurt) (m) 100% vegetable juices or **fruit juices**; vegetable drinks or **fruit drinks** sweetened with artificial sweeteners only (n) jams, beverage bases, and fruit curds or sauces that are unsweetened or artificially sweetened		(a) spices and herbs (b) fats and oils (c) plain cereal grains, pseudocereals (e.g., buckwheat, quinoa, amaranth), flour, pasta, rice, and plain cereal products; unsweetened potato chips (d) plain breads with minimal amounts of free sugars only used for activation of yeast in fermentation (<9 g/>1000 g pre-baking weight) (e) plain pastries (e.g., puff pastry, pastry dough) without fillings, and without dried fruits, nuts or chocolate (f) eggs and egg products (excluding egg-based desserts) (g) fresh, frozen, or cooked fruits, berries, vegetables (including salads without dressing) and root vegetables; unsweetened dried fruits (h) fruit and vegetables canned in 100% vegetable juice, or in artificially sweetened liquid (i) unsweetened nuts, seeds, coconut and coconut products (j) fresh meat, fresh fish, fresh seafood, tofu, and unsweetened legumes and mushrooms; mixed meat dishes without free sugars (k) coffee, tea and alcoholic beverages unsweetened or artificially sweetened (l) unsweetened milk and dairy products, and non-dairy milk substitutes (e.g., oat and soy drinks and yoghurt) (m) 100% vegetable juices; vegetable drinks sweetened with artificial sweeteners only (n) jams, beverage bases, and fruit curds or sauces that are unsweetened or artificially sweetened
Step 3	Food items in the food groups stated below are considered having minimal amounts of natural occurring sugars (<1%), and are assigned 100% of total sugars as added sugars:	Step 3	Food items in the food groups stated below are considered having minimal amounts of natural occurring sugars (<1%), and are assigned 100% of total sugars as free sugars:
	(a) confectionary except confectionary with dairy (e.g., chocolate, fudge); flavoured potato chips and other salty snacks (b) breakfast cereals and bars (e.g., muesli bars) without fruits, chocolate or dairy (c) sugar-sweetened ^1^ coffee and tea; beverage and soup bases (e.g., cordial, rosehip soup powder) with added sugars and without dairy; ice lollies (d) processed meats, fish, shellfish, blood products and vegetarian dishes including pies, filled pastries and breaded meats (e) sugar-sweetened ^1^ soda, sports drinks, flavoured water, and energy drinks without fruits (f) baked food items such as cookies, buns, donuts, sponge cake, and other batter based products without fruits, chocolate or dairy (g) sugar-sweetened ^1^ breads without fruits or dairy (h) sugar-sweetened ^1^ non-dairy milk substitutes (e.g., oat and soy drinks and yoghurt) without fruits (i) stock powder (j) table sugar and **syrups made from 100% added sugars** (k) sugar-sweetened ^1^ non-dairy alcoholic beverages and liqueurs (l) food items with added sugars that are pickled or cured (e.g., gravlax, pickled herring and pickled vegetables), or marinated; sushi rice (m) sauces, dressings, and mayonnaise that contain added sugars		(a) confectionary except confectionary with dairy (e.g., chocolate, fudge); flavoured potato chips and other salty snacks (b) breakfast cereals and bars (e.g., muesli bars) without fruits, chocolate or dairy (c) sugar-sweetened ^1^ coffee and tea; beverage and soup bases (e.g., cordial, rosehip soup powder) with free sugars and without dairy; ice lollies (d) processed meats, fish, shellfish, blood products and vegetarian dishes including pies, filled pastries and breaded meats (e) sugar-sweetened ^1^ soda, sports drinks, flavoured water, and energy drinks without fruits (f) baked food items such as cookies, buns, donuts, sponge cake, and other batter based products without fruits, chocolate or dairy (g) sugar-sweetened ^1^ breads without fruits or dairy (h) sugar-sweetened ^1^ non-dairy milk substitutes (e.g., oat and soy drinks and yoghurt) without fruits (i) stock powder (j) table sugar, **honey** and syrups (k) sugar-sweetened ^1^ non-dairy alcoholic beverages and liqueurs (l) food items with free sugars that are pickled or cured (e.g., gravlax, pickled herring and pickled vegetables), or marinated; sushi rice (m) sauces, dressings, and mayonnaise that contain free sugars (n) **fruit juice, fruit juice concentrate**
Step 4	Composite food items composed of food items with estimated added sugars values from steps 1–4. Composite food items were automatically calculated with a standardized recipe calculation method [15]	Step 4	Composite food items composed of food items with estimated free sugars values from steps 1–4. Composite food items were automatically calculated with a standardized recipe calculation method [15]
Step 5	Calculation of added sugars based on comparison with unsweetened variety [10]	Step 5	Calculation of free sugars based on comparison with unsweetened variety
	${\mathrm{AS}}_{100g}=\frac{100\;*\;\left(S_{\mathrm{us}}{\;-\;S}_{T}\right)\;}{S_{\mathrm{us}}\;-\;100}$ AS_100g_ = added sugars per 100g S_US_ = total sugars content per 100 g of unsweetened variety S_T_ = total sugars for the food that added sugars is to be estimated for		${\mathrm{FS}}_{100g}=\frac{100\;*\;\left(S_{\mathrm{us}}{-\;S}_{T}\right)\;}{S_{\mathrm{us}}\;-\;100}$ FS_100g_ = free sugars per 100g S_US_ = total sugars content per 100 g of unsweetened variety S_T_ = total sugars for the food that free sugars is to be estimated for
Step 6	Calculation of added sugars when analytical information is available on total sugars and individual intrinsic sugars (lactose and/or fructose)	Step 6	Calculation of free sugars when analytical information is available on total sugars and individual intrinsic sugars (lactose and/or fructose)
	(a) When analytical information is available on total sugars and lactose, and the food item does not contain fruits and/or vegetables: added sugars are the sum of total sugars minus lactose (b) When analytical information is available on total sugars and fructose, and the food item contains fruit and/or vegetables and are free from dairy: added sugars are the sum of total sugars minus fructose (c) When analytical information is available on total sugars, lactose and fructose, and the food item contains dairy, fruits and/or vegetables: added sugars are the sum of total sugars minus lactose and fructose		(a) When analytical information is available on total sugars and lactose, and the food item does not contain fruits and/or vegetables, **fruit juice, fruit juice concentrate or honey**: free sugars are the sum of total sugars minus lactose (b) When analytical information is available on total sugars and fructose, and the food item contains fruit and/or vegetables, and are free from dairy, **fruit juice, fruit juice concentrate or honey**: free sugars are the sum of total sugars minus fructose (c) When analytical information is available on total sugars, lactose and fructose, and the food item contains dairy, fruits and/or vegetables, **and are free from fruit juice, fruit juice concentrate and honey**: free sugars are the sum of total sugars minus lactose and fructose
Step 7	Sugars values are borrowed from similar food items that are previously known (from step 1–6 or other databases, preferably within the Swedish food composition database)	Step 7	Sugars values are borrowed from similar products that are previously known (from step 1–6 or other databases, preferably within the Swedish food composition database)
	(a) Added sugars value is taken directly from similar food item (b) Added sugars value is estimated based on information on similar food item		(a) Free sugars value is taken directly from similar food item (b) Free sugars value is estimated based on information on similar food item
Step 8	Subjective estimation based on available information (from ingredients lists or common recipes), where added sugars values are calculated in accordance to the proportions of the reference information. First added sugars content is calculated within the reference food item. The same proportion of added sugars is calculated for the estimated food item.	Step 8	Subjective estimation based on available information (from ingredients lists or common recipes), where free sugars values are calculated in accordance to the proportions of the reference information. First free sugars content is calculated within the reference food item. The same proportion of free sugars is calculated for the estimated food item.
Step 9	Composite food items composed of food items with estimated added sugars values from previous steps, where added sugars content of any of the food items included was determined by using steps 5–10, i.e., step 4 is repeated with more food items available	Step 9	Composite food items composed of food items with estimated free sugars values from previous steps, where free sugars content of any of the food items included was determined by using steps 5–10, i.e., step 4 is repeated with more food items available
Step 10	When steps 1–9 are not applicable, added sugars are assigned 50% of total sugars. Consider the plausibility of the food item’s estimated added sugars content, and make sure no previous steps are applicable	Step 10	When steps 1–9 are not applicable, free sugars are assigned 50% of total sugars. Consider the plausibility of the food item’s estimated free sugars content, and make sure no previous steps are applicable

^1^ Sweetened with refined sugars or isolated sugar preparations added to the product. Food items categorized differently between added and free sugars are marked in bold.

## Appendix B

Modifications made in classification towards methods described by Louie et al. and Kibblewhite, Nettleton et al. are presented in Table A2. Step numbers and descriptions are not identical to referenced methods. Added sugars classifications were compared with definitions by Louie et al. Free sugars classifications were compared with definitions by Kibblewhite, Nettleton et al.

**Table A2:** Discrepancies in added and free sugars classification between our procedure and methods described by Louie et al. [10], and Kibblewhite, Nettleton et al. [11].

Step	Step Explanation (number in parenthesis indicate reference step number assignment)
*Step 2*	*Food item is included in one of the groups meeting the criteria for having no added sugars*
2 (a)	Honey was excluded as added sugars, and included within this step
2 (c)	Pseudocereals (e.g., buckwheat, quinoa) was added Oats was removed as separate step (2 (p)), and included within this step Plain cereal products was added Unsweetened potato chips was added
2 (d)	Clarification added; With minimal amounts of added sugars (<9 g/>1000 g pre-baking weight)
2 (e)	Puff pastry and pastry dough were added
2 (g)	Root vegetables was added Berries was added Frozen, and cooked were added Unsweetened dried fruits was removed as separate step (2 (s)), and included within this step Meats, fish, seafood, and tofu (2 (f)) were removed from this step
2 (h)	Vegetables canned in 100% vegetable juice was added
2 (j)	Meats, fish, seafood, and tofu were included within this step (2 (f)) Legumes was removed as separate step, and included within this step (2 (i)) Mushrooms was added
2 (k)	Coffee, tea, and alcoholic beverages were combined within this step, instead of having separate steps (2 (k), 2 (l))
2 (l)	Dairy products was removed as separate step (2 (n)) and included within this step Non-dairy milk substitutes (e.g., oat and soy drinks and yoghurt) without added sugars was added Breast milk was removed (2 (m))
2 (n)	This step was added; Jams, beverage bases, and fruit curd or sauce that are unsweetened or sweetened without added sugars
*Step 3*	*Food item is included in one of the groups meeting the criteria for having minimal intrinsic sugars and where 100% of sugars are added*
3 (a)	Flavoured potato chips and salty snacks were added
3 (c)	Soup bases was added Ice lollies was added
3 (d)	Processed and breaded meats were combined within this step, instead of having separate steps (3 (d), 3 (e)) Fish and shellfish were added Blood products was added Vegetarian dishes was added
3 (g)	This step was added; Sugar-sweetened breads without fruits or dairy
3 (h)	Non-dairy milk substitutes was added as term to include different kinds of non-dairy substitutes for milk (e.g., oat and soy drinks (3 (h)) and yoghurt)
3 (k)	This step was added; Sugar-sweetened non-dairy alcoholic beverages and liqueurs
3 (l)	This step was added; Foods with added sugars that are pickled (e.g., cured salmon, pickled herring, beets), cured, or marinated; sushi rice
3 (m)	This step was added; Sauces, dressings, and mayonnaise that contain added sugars
*Step 4*	*The food item is composed by a standard recipe and added sugars content of all ingredients known by previous steps 1–3*
4	The calculation method used in this step was changed
*Step 6*	*Food item has available analytical data of individual intrinsic sugars (lactose, fructose)*
6 (b)	This step was added; When analytical information is available on total sugars and fructose, and the food item contains fruit and/or vegetables and are free from dairy: added sugars are the sum of total sugars minus fructose
6 (c)	This step was added; When analytical information is available on total sugars, lactose and fructose, and the food item contains dairy, fruits and/or vegetables: added sugars are the sum of total sugars minus lactose and fructose
*Step 7*	*A comparable food item with known added sugars content estimated by steps 1-6 are available*
7 (a) (b)	This step was divided into two categories (7); (a) Added sugars value is taken from similar food item (b) Added sugars value is estimated based on information on similar foods
*Step 8*	*Added sugars can subjectively be estimated by available information (ingredient list or recipe)*
8	The estimation method was modified; Added sugars calculated in accordance to the proportions of the reference information. First added sugars content is calculated within the reference food item. The same proportion of added sugars is calculated for the estimated food item.
*Step 9*	*A standard recipe has been used with known added sugars content of all food items after using steps 5–10*
9	Added sugars values estimated according step 10 was included The calculation method used in this step was changed
*Step 2*	*Food item is included in one of the groups meeting the criteria for having no free sugars*
2 (c)	Pseudocereals (e.g., buckwheat, quinoa) was added Plain cereal products was added Unsweetened potato chips was added
2 (d)	Clarification added; With minimal amounts of added sugars (<9 g/>1000 g pre-baking weight)
2 (g)	Vegetables was added Root vegetables was added Berries was added Frozen, and cooked were added Fruits canned in syrup, sweetened with artificial sweetener (2 (g)) was removed from this step and formed a new step
2 (h)	This step was added Vegetables canned in 100% vegetable juice were added
2 (i)	Coconut and coconut products were added
2 (j)	Fish was added Mushrooms was added Mixed meat dishes without free sugars was added
2 (l)	Non-dairy milk substitutes (e.g., oat and soy drinks and yoghurt) without free sugars was added
2 (m)	This step was added; 100% vegetable juices; vegetable drinks sweetened with artificial sweeteners only
2 (n)	This step was added; Jams, beverage bases, and fruit curd or sauce that are unsweetened or sweetened without free sugars
*Step 3*	*Food item is included in one of the groups meeting the criteria for having minimal intrinsic sugars and where 100% of sugars are free*
3 (a)	Without dairy was added Flavoured potato chips and salty snacks were added
3 (c)	Tea was added Soup bases was added Ice lollies was added
3 (d)	Fish was added Blood products was added Vegetarian dishes was added
3 (e)	Without fruits was added
3 (g)	This step was added; Sugar-sweetened breads without fruits or dairy (not restricted to gluten free (3 (h)))
3 (h)	Non-dairy milk substitutes was added as term to include different kinds of non-dairy substitutes for milk (e.g., oat and soy (3 (m)) drinks and yoghurt)
3 (j)	Honey was added
3 (k)	This step was added; Sugar-sweetened non-dairy alcoholic beverages and liqueurs were added
3 (l)	This step was added; Foods with added sugars that are pickled (e.g., cured salmon, pickled herring, beets), cured, or marinated; sushi rice
3 (m)	Mayonnaise based sauces was added Pasta sauces was excluded from this step (3 (k))
3 (n)	Fruit purées was excluded as free sugars (3 (d)) Jams (3 (d)) was excluded from this step due to a major part often being fruit purées
*Step 4*	*The food item is composed by a standard recipe and free sugars content of all ingredients known by previous steps 1* *–* *3*
4	The calculation method used in this step was changed
*Step 5*	*Calculation of free sugars based on comparison with unsweetened variety*
5	Change in formula
*Step 6*	*Food item has available analytical data of individual intrinsic sugars (lactose, fructose)*
6 (a)	This step was considerably modified; When analytical information is available on total sugars and lactose, and the food item does not contain fruits, fruit juice, fruit juice concentrate or honey: free sugars are the sum of total sugars minus lactose (6)
6 (b)	This step was added; When analytical information is available on total sugars and fructose, and the food item contains fruit and/or vegetables, and are free from dairy, fruit juice, fruit juice concentrate or honey: free sugars are the sum of total sugars minus fructose
6 (c)	This step was added; When analytical information is available on total sugars, lactose and fructose, and the food item contains dairy, fruits and/or vegetables, and are free from fruit juice, fruit juice concentrate and honey: free sugars are the sum of total sugars minus lactose and fructose
*Step 7*	*A comparable food item with known free sugars content estimated by steps 1–6 are available*
7 (a) (b)	This step was divided to two categories (7); (a) Free sugars value is taken directly from similar food item (b) Free sugars value is estimated based on information on similar foods
*Step 8*	*Free sugars can subjectively be estimated by available information (ingredient list or recipe)*
8	The estimation method was modified; Free sugars calculated in accordance to the proportions of the reference information. First free sugars content is calculated within the reference food item. The same proportion of free sugars is calculated for the estimated food item.
*Step 9*	*A standard recipe has been used with known free sugars content of all food items after using steps 5* *–* *10*
9	The calculation method used in this step was changed
